# The Role of the Visual Versus Verbal Modality in Learning Novel Verbs

**DOI:** 10.3390/children12060722

**Published:** 2025-05-31

**Authors:** Maria Luisa Lorusso, Laura Pigazzini, Laura Zampini, Michele Burigo, Martina Caccia, Anna Milani, Massimo Molteni

**Affiliations:** Scientific Institute IRCCS E. Medea, Via Don Luigi Monza 20, 23842 Bosisio Parini, LC, Italy; laura.pigazzini@compitipoint.it (L.P.); laura.zampini1@unimib.it (L.Z.); michele.burigo@gmail.com (M.B.); martina.caccia123@gmail.com (M.C.); anna.milani@lanostrafamiglia.it (A.M.); massimo.molteni@lanostrafamiglia.it (M.M.)

**Keywords:** abstract concepts, verb learning, verbal modality, visual modality, embodiment

## Abstract

Background/Objectives: Verbs are considered to be more abstract than nouns, as they represent actions, states, and events, which are less tangible, more flexible in their meaning and thus less univocally specified. It has been suggested that children acquire abstract concepts based on their linguistic contexts of use, making use of semantic and syntactic cues. By contrast, according to theories of embodied cognition, conceptual knowledge is based on physical and perceptual interaction with the world. The present study investigates whether the verbal and the visual modality produce similar or different results in the processes of construction and reactivation of novel verbs, corresponding to new compositional abstract concepts, in children of different ages. In Experiment 1, the acquisition of the concept was determined based on the quality of verbal explanation; in Experiment 2, participants were asked to decide whether a visual representation fitted the concept or not. Thus, response modality could be either explicit or implicit, and either congruent or incongruent with respect to learning modality. Methods: In Experiment 1, 100 children from grade 1 to 5 were asked to explain the meaning of verbs introduced via verbal or visual instances. In Experiment 2, 15 children aged 8 to 10 had to judge pictures as (not) being examples of previously verbally or visually presented novel verbs. Results: The results of Experiment 1 show more accurate explanations after verbal presentation across all grades. In Experiment 2, verbal presentation was no longer associated with more accurate matching responses, but rather with slower decision times. Conclusions: Modality congruence, explicitness and linguistic (semantic and syntactic) factors were all shown to play a role, which is discussed in a developmental perspective.

## 1. Introduction

The distinction between concrete and abstract concepts is an ontological distinction (i.e., a distinction in the relationships between fundamental concepts that support and structure human representations of knowledge) based on whether they refer to something that can be perceived and acted upon or to something that is only internally represented [1].

Although children are exposed to a continuous stream of information, they learn very early to detect regularities (i.e., some characteristics that are frequently linked together in an object) and to integrate them into semantic clusters (networks representing the relationships between these characteristics); the product of this process is the concept, which simplifies and systematizes information deriving from the real world [2]. Since sensory experience seems to be the starting point of concept formation, it is not clear how toddlers and preschoolers could learn abstract concepts (e.g., happiness, freedom, life, etc.) that are not directly experienced through the senses and that have no concrete referents in the real world. The studies comparing the acquisition of abstract and concrete concepts found a significant advantage in processing concrete words in different cognitive tasks: this “concreteness effect” could be explained as the result of a dual representation, in both the verbal and the imaginal system, for concrete concepts [3,4,5], whereas abstract concepts would only be coded in the verbal modality, or as the result of a greater availability of contextual information for concrete words [6,7].

While the meaning of concrete concepts is acquired by observation and analysis of the extralinguistic context, in a word-to-world pairing, the acquisition of abstract concepts, which have no concrete referents in the real world, has been suggested to be based on the consideration of their linguistic contexts of use, in a structure-to-world pairing [8,9]. In addition, children may use the syntactic frames or structures in which verbs are presented as a source of information about their meaning, therefore a series of “syntactic bootstrapping” mechanisms [9,10,11,12] would be necessary for semantic development. According to Gleitman’s proposal [9], syntactic bootstrapping provides a way through which children could learn “hard” words, whose meanings can hardly be perceived, while “easy” words, by contrast, refer to concrete words that can be learned observing the surrounding world. In fact, the focusing of attention on certain aspects of syntactic structure could restrict the possible meanings of a concept [13]. Also following Chomsky’s perspective [14], novel word acquisition is guided by the innate ability of the human brain to acquire grammatical categories and syntactic rules. Even in more recent contributions, Chomsky proposes a syntax-based view of language acquisition and mastery, suggesting that natural language consists of ‘internalist computations’ [15]. Many studies, in accordance with selectional restrictions [14,15,16,17,18], have shown that toddlers are capable of using a verb’s syntactic structure to construct a representation of its meaning [19]. For instance, 25-month-old toddlers were found to look longer at a transitive action video when a novel verb was presented in a transitive sentence structure, and at an intransitive action video when the novel verb was presented in an intransitive structure. In other words, the toddlers were using their knowledge of syntax to deduce the meaning of new words [12]. Furthermore, 26- and 30-month-olds presented with two images (e.g., a cookie and a book) seem to be faster to fixate on the cookie when they hear a sentence such as “Eat the cookie” than the more generic “Take the cookie” [20]. This suggests that they are able to use the argument structure of the verb (where the probability of having a cookie as the argument of “eat” is higher than having a cookie as the argument of “take”) to access the meaning of the noun. According to [21], infants as young as 18 months use their representations of known verbs to inform (and start representing) the meaning of a novel noun that appears as its argument. It could also be hypothesized that meanings arise from the relationships between different terms in the same linguistic context even in the absence of any information about syntactic properties [22,23], since words which are often co-occurrent in a sentence are also conceptually related. Moreover, recent evidence demonstrated that simply hearing a novel verb in a sentence, without an accompanying scene, helps toddlers to infer some information about its meaning, and to use that information later to find an appropriate referent when a visual scene becomes available [24].

By contrast, according to theories of embodied cognition, conceptual knowledge is based on the interaction of a physical body with the world [25], and this applies to the acquisition of abstract concepts as well. Recent studies investigating sentence comprehension [26] have found an activation of the brain sensorimotor areas with both concrete and abstract language input. The activation of these areas during the comprehension of abstract concepts has been interpreted as a demonstration of the existence of general action schemata, which are devoted not only to the concrete domain. These schemata originate from repeated observations of concrete actions but they can subsequently be generalized to actions referred to the same schema in an abstract situation. For instance, the action schema developed for the “transfer of goods” in a concrete context can also be used to symbolize the “transfer of information” in the abstract context of communication (e.g., to pass an idea, to exchange opinions, etc.). Thus, abstract knowledge would be closely related both to images and to motor schemata [27,28].

The two hypotheses clearly imply that different kinds of information are crucial to learn abstract concepts: linguistic input vs. visual input.

Verbs surely represent a particularly interesting category of words. It is often assumed that verbs are abstract by definition, since they “take a perspective on events that cannot be predicted from observing those events and understanding human intentions” ([11], p. 268). Following [15], we assume that semantic structures of verbs reflect the non-linguistic conceptual representations that are used to represent events. Both kinds of representations, in fact, require distinguishing between predicates and arguments, and thus between relations and the elements in the relation [29,30]. Many studies investigating verb acquisition employed verbs (often substituted by pseudowords) referring to already acquired concepts, i.e., even when the words were unknown, the underlying concept was not (e.g., [31]). This is indeed consistent with accounts of verb learning assuming that the concept expressed by the verb must be acquired before the verb can be learned [32]. Nonetheless, it is also true that in real life situations children have to learn completely novel verbs, referring to relations that are probably not completely unknown, but which very often focus on aspects of the relation that the children had not considered before. In other words, novel verbs often refer to concepts that are conceivable, but possibly not conceived as a single concept yet. At a closer look, probably every new verb which is learned has the function to express with a single word what was previously encoded by whole sentences. This is also the case of adults learning new terms from specific or technical language (e.g., “leasing” and “franchising” in financial vocabulary, “bisecting” in experimental psychology, etc.) or words in foreign languages which have no direct translation in one’s own language (e.g., “to mourn” in English, which would correspond to “to feel or express sadness for the death of a loved one” in most roman languages). These verbs probably lie in the intermediate range of abstractness, since they encode very specific information concerning not only the relation (the action) but also its argument. The relevant advantage of these “specification verbs” is that they are more representative of ecologically plausible learning situations. A thorough description of the processes involved in learning novel verbs in foreign languages is provided by recent studies [33,34,35], highlighting that the ease with which verbs in a new language are learned depends, among other variables, on the degree of complexity characterizing verb morphology in the speaker’s L1 and L2, with less morphological inflection corresponding to earlier and better learning. Linguistic variables such as morphology, syntax, phonology and lexical complexity, in turn, can be influenced by age in different ways.

The aim of the present study is to investigate whether the verbal and the visual modality produce similar or different results in the processes of construction and reactivation of novel verbs corresponding to new compositional abstract concepts in children of different ages and in a morphologically rich language such as Italian.

Published studies about the (free or cued) recall or the recognition of previously presented stimuli (words or pictures) have reported a so-called “picture superiority effect”, suggesting an advantage of the visual modality for long-term memorization [36,37]. This line of research would support the prediction that visual presentation leads to better performance, since novel concepts need to be memorized to be subsequently recalled, comprehended and used. Specifically, in an alternative account partially opposed to dual-coding theories [5], it has been proposed that the advantage of the visual modality is intrinsic to semantic organization [38], so that access to semantic features is faster and more direct from pictures than from words [39], or that categorization is faster with visual input modality [40]. However, very few studies have addressed such issues in children, and mixed results have generally been reported [41]. More crucially, those studies concern memory, while the focus of interest for the present study goes far beyond memorization, encompassing concept formation and concept reactivation; further, we were interested in the distinction between concrete and abstract concepts, and the distinction between different types of verb, such as action and mental verbs, that were not addressed by the mentioned studies. Indeed, some verbs are considered to be particularly abstract because they refer not to physical actions (unlike action verbs) but rather to psychological states (such as think, believe, fear or imagine), and it was shown that verbal descriptions are more effective (or better, the only effective ones) for young children’s induction of verbs expressing mental states, while pictures are most effective in introducing action verbs [9]. The present study will investigate the effectiveness of the two modalities in inducing the meaning of novel verbs, including mental verbs and action verbs. Actually, the most recent trends in the study of abstract versus concrete concepts highlight the importance to distinguish among different types of words even more than between abstract and concrete ones [42,43].

A further element of interest is the age range of the participants, six to ten years old, covering the whole primary school cycle. Indeed, the children participating in the study are generally older than the children in most other studies on concept acquisition. This choice is due to the tasks requiring that the children are able to fully grasp the kinds of notions proposed in the learning situations, and to understand complex language and interpret complex visual scenes, which would be representative of real-life and school-learning situations. This excludes participants at very early stages of cognitive development, but at the same time it has interesting implications for educational practice in school-age years. Several studies from the field of educational psychology point to the positive role of visual information to complement verbal instruction at school, taking into consideration various school levels (e.g., [41,44]). However, to our knowledge, most of these studies compare verbal-only instruction with verbal and visual instruction, and none of them has directly compared purely verbal with purely visual instruction. The age range covered by the study, moreover, is characterized by great changes in inferential abilities and information-processing skills, so that significant changes could be expected in the way children face instance-based learning situations.

In Experiment 1, a concept learning task has been proposed to children of different ages, by presenting verbal vs. visual descriptions of the novel concept, through two subsequent presentations. The comparison of the explanations of the concepts provided by the children after presentation in the two modalities indicates whether linguistic or rather visual–perceptual information facilitates the acquisition of novel verbs (and, possibly, of abstract concepts in general).

The administration of two subsequent presentations of the set of positive and negative instances of each concept allows investigation of learning effects induced by repetition (see [45,46]). It could be hypothesized, in fact, that the time-course of learning differs in the two modalities, for instance modifying or even reversing the pattern of results found after the first presentation, and that such changes may be specific to certain age groups (see for instance [41]).

Experiment 2 was specifically designed to test the hypothesis that modality congruence between presentation and task requirements would be the basis for the advantage of the verbal modality found in Experiment 1. Due to the impossibility to ask for a description of the concept in the visual modality (a drawing would be too difficult and too much dependent on visual-motor skills not reflecting real understanding of the concept), a perfect equivalent of Experiment 1 could not be designed, and a judgment task was chosen instead. The request being to judge for a series of pictures which ones did and which did not represent the novel concept, it was expected that any effect due to modality congruence would have favored the concepts that had been presented in the visual rather than the verbal modality.

## 2. Materials and Methods

### 2.1. Participants

One hundred children, ranging from 6 to 10 years, participated in Experiment 1. Twenty participants for each grade, from the first to the fifth, were recruited in local primary schools; in order to control for the influence of specific curricula included in the school programs, the participants were selected from different classrooms of different grades in six different schools.

Fifteen children, recruited in two local primary schools and aged 8 to 10 years (M = 9.03; SD = 0.8), participated in Experiment 2. The age range was more restricted as compared to Experiment 1, as no differences due to age or grade had emerged from the first study.

All participants were monolingual native Italian speakers and they were regularly attending school; none of the children suffered from developmental disabilities, such as linguistic impairments, learning disabilities or perceptual and motor problems, as determined from school records. All parents signed informed consent after the project had been presented to, and approved by, the school boards. The study was approved by the Ethics Committee of the Scientific Institute E. Medea according to standards of the Helsinki Declaration (1964).

### 2.2. Materials

Ten novel abstract concepts were created and used in both Experiment 1 and Experiment 2 (see Appendix A), each one combining two existing concepts, in order to create a single concept for which no single Italian word is available. For each concept, a completely new label was coined. For example, the concept “to do a good deed to an elderly person” was constructed from two constituent elements, to help and elderly person, and the lexical label “ging-ere” (a pseudoword formed by a non-existing lexical root and a verb infinitive inflection) was assigned to this new concept. The novel concepts were judged for their level of abstractness, on a seven point Likert scale ranging from “fully abstract” (1) to “fully concrete” (7), by twelve Italian native speaker adults; all the concepts selected for the present study were rated as having a score lower than 4 with an agreement between independent judges of at least 10/12.

Six representative situations of each concept were created: four were positive instances of the concept (i.e., situations depicting both the constituents of the new concept), and two were negative instances (i.e., situations in which at least one of the two constituents was absent). For each concept, two different presentation modalities were prepared. In the visual modality, each concept was represented by six different pictures (black-and-white line drawings), whereas in the verbal modality a linguistic description of each picture was audiotaped. In order to ensure that each verbal description contained all the elements included in the corresponding picture, twelve adults were asked to rate the equivalence between the two representations (visual and verbal) of the same concept on a 1 to 100 scale (per cent). After each judgment, the descriptions and drawings (see examples in Appendix B) were adapted taking into account the judges’ suggestions. The mean percentage of agreement between the two modalities for the final versions was 88%.

All instances (verbal descriptions and visual scenes) were accompanied by an auditory presented sentence in which the novel verb appeared. However, since all verbs were used intransitively (with no direct object on which the action is transferred to), only the subject and the verb in its progressive form were presented, so that syntactic bootstrapping mechanisms for inferring verb meaning were kept to a minimum in all conditions.

### 2.3. Procedure

The stimuli were presented on a computer screen using E-Prime software (version 2.0, PST, Pittsburgh, PA, USA). All participants were individually tested in a quiet room at their school. The experimental design was essentially within-subject. Each participant was presented with both visual and verbal stimuli: 50 children (10 from each class) were first exposed to five concepts in the visual modality and then to five different concepts in the verbal modality, whereas the remaining 50 participants were exposed first to the verbal and then to the visual modality. The presentation modality and the serial position of the different concepts were counterbalanced among the participants. Specifically, two different presentations of the concept were prepared, A and B: in presentation B, the modality of presentation was reversed with respect to presentation A, so that all concepts that had been presented in the visual modality in presentation A were presented in the verbal modality in presentation B and vice versa. The two presentation series were administered to half of the participants on each grade: so, 10 children in each grade received presentation A and 10 children received presentation B. The order of presentation within each series was also varied, so that all the children in each grade received the concepts in a different order.

For each concept, the picture remained on the screen for a time equivalent to the duration of the corresponding audiotaped verbal description.

The six situations (either pictures or verbal descriptions depending on the condition) conveying the meaning of the novel concept were presented in a fixed sequence. In the visual modality, while the participants were looking at a picture, they heard a voice stating whether the agent in the picture was or was not performing the action representing the novel concept. For instance, for the concept gingere (“ging” + infinitive inflection, to do a good deed to an elderly person), when the child was looking at a picture representing a boy helping his grandmother to set the table, the voice said “il bambino sta gingendo” (the boy is “ging”-ing), whereas when the child was looking at a picture depicting a boy helping another boy, the voice said “il bambino non sta gingendo” (the boy is not “ging”-ing). As to the verbal modality, the participants heard a voice reading the description and then labeling the concept in a positive or negative form (e.g., “The boy is helping his elderly grandmother to set the table. The boy is “ging”-ing vs. “The boy is helping another boy to set the table. The boy is not “ging”-ing).

In Experiment 1, after the six situations had been presented, the children were asked to explain the meaning of the novel concept. All answers were audiotaped and transcribed. No time limits were given. After the child’s answer, the same concept was presented for the second time in the same modality and the child was asked again about its meaning. Then, a new concept was introduced. The mean duration of each experimental session was about 40 min.

As to Experiment 2, the stimuli were presented on a computer screen following the same procedure described before. Each participant was presented with both visual and verbal stimuli: eight children were first exposed to five concepts in the verbal modality and then to five concepts in the visual modality, whereas the remaining seven participants were first exposed to the visual and then to the verbal modality.

After the six situations were presented in either the visual or the verbal modality, the children were presented with three new pictures in random order: two of them depicted the concept and one did not. Each picture was anticipated by a vocal question asking the child if the image was an instance of the newly learned concept or not; for example, after the situations representing the concept “pobeare” (“pobe” + infinitive marker, i.e., to reach an object using another object) had been shown, the child was presented with a question like “il nonno sta pobeando?” (is grandfather “pobe”-ing?) followed by a picture of a man reaching the TV remote control using his walking stick. The child responded by pressing two different buttons (corresponding to “yes” and “no”) on a response box (two different mappings of the response keys, balanced among subjects, were used to prevent handedness effects). No time limits and no feedback on accuracy were given to the child. Accuracy scores and decision times (from the moment in which the figure appeared) were recorded for each question. Response times below 200 ms (n = 1) were considered spurious responses and were excluded from subsequent analyses. Each concept was presented once, through the visual modality for half of the participants and through the verbal modality for the remaining half. The mean duration of each session was about 20 min.

### 2.4. Scoring

In Experiment 1, the definitions given by the participants were coded according to different procedures.

First, each definition was given a score ranging from 0 to 2: 0 was assigned to completely wrong answers (i.e., the mere repetition of an example or a definition that does not report any of the constituent elements of the concept); 1 was assigned to definitions including at least one of the two constituent elements of the concept; 2 was assigned to definitions reporting both constituent elements. If the definition was unclear or it did not indicate full comprehension of the concept, a penalty of 0.5 points was applied; however, linguistic inaccuracy or expressive difficulties were not penalized. For instance, definitions like “to help people” or “to do a good deed” for the concept *gingere* (to do a good deed to an elderly person, formed by the two conceptual elements: helpful action + elderly person) were assigned 1 point, as they contain one of the two constituent elements of this concept; by contrast, a too-generic definition, like “to do a right thing”, was assigned 0.5 points and a description like “to help grandparents to take something”, which contains both elements, but refers to a too specific situation, was assigned 1.5 points. The sum of the scores obtained for each definition constitutes the Total Score of each child.

All answers were coded by two independent raters. Inter-coder reliability (two independent coders) for the scoring was 0.92 (Cohen’s Kappa).

In Experiment 2, accuracy scores directly represented the number (expressed as percentage) of correct judgments for the five instances presented for each of the concepts.

## 3. Results

Data from Experiment 1, requiring children to respond in the verbal modality, were analyzed with GLM (repeated-measures ANOVA) on mean Total scores using SPSS software (version 20). First of all, the two presentation series A and B (where the same concepts were presented but presentation modality was reversed) were compared, showing no significant difference (*p* > 0.9) and no interaction with Grade (*p* > 0.8). Therefore, the two presentation series were collapsed in the following analysis, providing twenty mean verbal and twenty mean visual scores (each one based on the presentation of five concepts), collected from the same group of twenty participants, for each grade. The within-subject factors taken into consideration were Modality (verbal vs. visual) and Repetition (first vs. second presentation), while Grade (1, 2, 3, 4, 5) was entered as a between-subject variable. A sensitivity analysis (G-Power) revealed that 100 participants in a repeated-measures (four measures with an average correlation of 0.7 and 5 groups) analysis are sufficient to highlight a within-subject difference with an effect size of f = 0.09 (medium), a between-subject difference with an effect size of f = 0.31 (medium), and a within–between-subject interaction corresponding to a small-to-moderate effect size (f = 0.116) with a power of 0.80.

The analysis reveals a main effect of Modality (F (1, 95) = 5.652; *p* = 0.019, η_p_^2^ = 0.054) and Grade (F (4, 95) = 13.330; *p* < 0.001, η_p_^2^ = 0.359, with no significant interactions between Modality and Grade.

**Figure 1:**
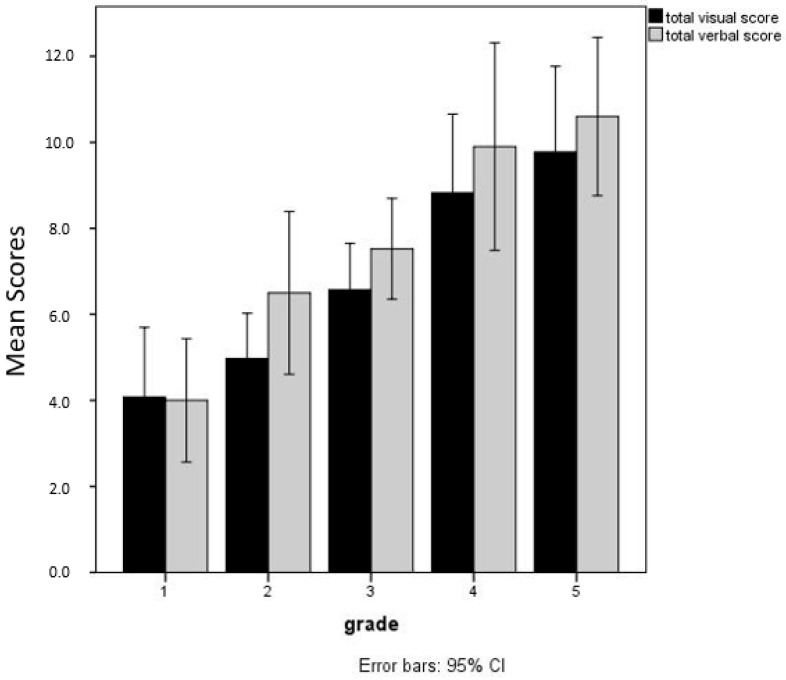
Mean total scores obtained for the descriptions given after presentation in the visual and verbal modality, for each grade.

Post hoc tests (Tukey’s test assessing the significance of differences between pairs of group means) show that presentation in the verbal modality leads to better descriptions of the concept than presentation in the visual modality and this advantage is significant from the second grade onwards, as can be seen in Figure 1. Furthermore, total scores differ significantly between non-consecutive grades (i.e., with a temporal gap of two years).

**Figure 2:**
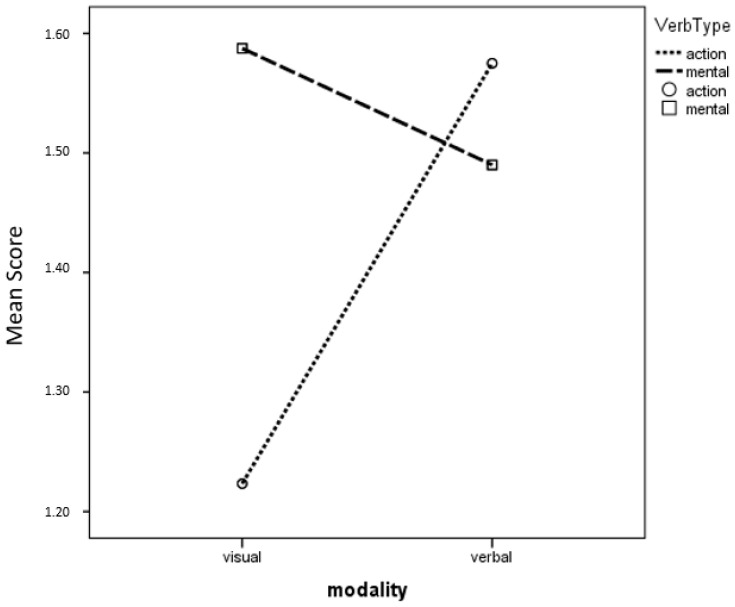
Verb Type by Modality Interaction. Mean scores for action and mental verbs, presented in the visual and verbal modality.

As expected, a main effect of Repetition also emerges, expressing learning effects across the first and second presentation of the same concept (F (1, 99) = 23.293; *p* < 0.001, η_p_^2^ = 0.190). However, the learning effect is not affected by presentation modality (interaction between Repetition and Modality, *p* > 0.05). Finally, since previous studies [9] had suggested different acquisition pathways for mental verbs (better acquired through the verbal modality) and for action verbs (better acquired through the visual modality), the concepts (see Appendix A) were subdivided according to verb Type, with scores obtained from verbs relating to mental states or processes (caglistare, dopanire, difulare, sofanire) averaged to express a “mental verb” score, and scores from verbs indicating more concrete actions (dortare, frintolare, gingere, mupergere, pobeare, suntorare) averaged to express an “action verb” score. A repeated-measures ANOVA with verb Type (mental verbs versus action verbs) and Modality as within-subject factors and Grade as a between-subject factor revealed an advantage for mental verbs (main effect of verb Type, F(1, 95) = 4.809, *p* = 0.031, η_p_^2^ = 0.048) and an interesting interaction of Modality with verb Type, F (1, 98) = 14.411; *p* < 0.001, η_p_^2^ = 0.128. At post hoc analyses, it was found that action verbs benefit from verbal more than visual presentation, while mental verbs yield similar results in the two modalities (see Figure 2).

**Figure 3:**
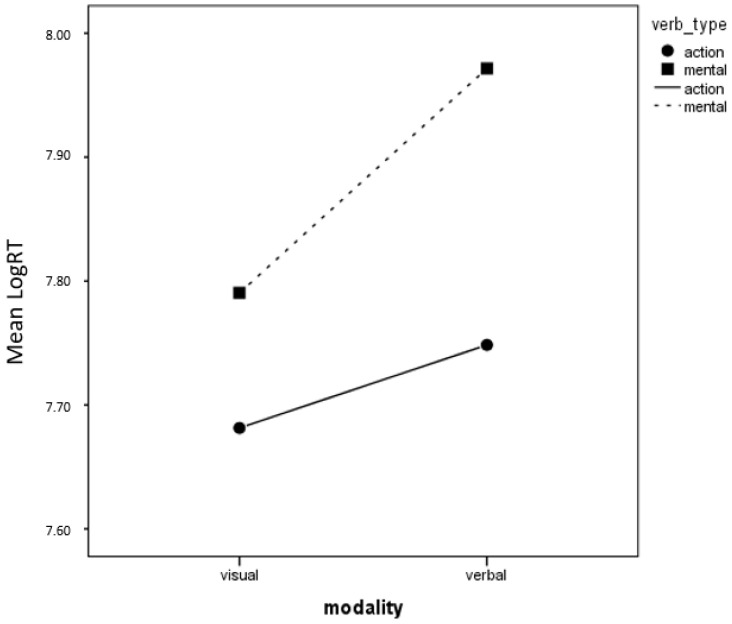
RTs (Logarithmic transformation) for visual representations of novel verbs, for the two verb types, after presentation in the verbal and in the visual modality.

Coming to Experiment 2, where responses were given in the visual modality, a GLM analysis including modality and verb type as fixed factors and subject as a casual factor shows no significant differences in accuracy of judging the pertinence of each picture to represent a concept in the two conditions, verbal and visual (verbal, M = 10.80, SD = 2.37; visual, M = 10.73, SD = 2.15; F (1, 13.007) = 0.260; *p* > 0.05, η_p_^2^ = 0.020). Conversely, participants are faster (response times with logarithmic transformation) when they have to judge pictures presented after visual than after verbal presentation (F (1, 13.821) = 11.254; *p* = 0.005, η_p_^2^ = 0.449). Also, responses to action verbs are faster (F (1, 13.897) = 14.367, *p* = 0.002, η_p_^2^ = 0.508) but less accurate (F (1, 13.022) = 8.422, *p* = 0.012, η_p_^2^ = 0.393) than responses to mental verbs, but no interactions with Modality emerge. Response times for the two modalities and for the two verb types are shown in Figure 3.

The analysis of a further distinction, between positive and negative instances of the concept (repeated-measures ANOVA with two within-subject factors, modality and instance type and age as a covariate) highlighted a significant difference between positive and negative instances of the concept, for accuracy measures (percent correct responses) only (F(1, 13) = 9.027, *p* = 0.010, η_p_^2^ = 0.410). More precisely, responses to positive instances were more accurate than responses to negative instances. Moreover, a significant interaction with age emerged (F(1, 13) = 6.810, *p* = 0.022, η_p_^2^ = 0.344), indicating that accuracy in responses to negative instances increases with age more than accuracy in responses to positive instances. However, no interactions with Modality emerged. Indeed, sensitivity analysis revealed that 15 participants in a repeated-measures (4 measures with an average correlation of 0.7 and a single group) analysis are sufficient to highlight a within-subject difference corresponding to an effect size of f = 0.244 (medium), with a power of 0.80. Nonetheless, any interactions with modality were far from significance.

## 4. Discussion

The results of the first experiment showed a significant advantage of the verbal presentation modality over the visual one. This advantage, moreover, seems to be independent of the age of the children and of the order of presentation, suggesting a general, very pervasive effect not specific of a developmental stage and not modulated by repetition. This finding lends support to the hypothesis that language is the main vehicle for the acquisition of new abstract concepts [6,7,8,9,10,11,12]. Since repeated presentation of information does not modify the advantage of the verbal over the visual modality, the advantage appears to be a substantial rather than a transitory one. This result is not in line with the predictions from studies on the “picture superiority effect” showing an advantage of the visual presentation in various memory tasks, even if no time constraints are present (claimed in [44] to be a condition for the picture superiority effect) and deeper, conceptual processing is required (suggested to be crucial by [36]). Moreover, a reversed-picture superiority effect (i.e., an advantage of the verbal over visual modality) is observed although no perceptual processing of words is requested, being an oral task (a condition shown by [47] to lead to the reversal of the effect). It is thus evident that memory processes are not the only ones at play and that concept formation is constrained by different processes than simple item recognition or recall.

There are, indeed, a number of alternative hypotheses that should be considered. First of all, the task in experiment 1 was a specifically linguistic one, requiring the children to verbally describe their understanding of what the newly introduced concept might be. This means that the advantage of the verbal presentation modality could depend on a verbal description being facilitating in view of the request to give another verbal description, i.e., the advantage itself could be interpreted as an effect of the congruence between presentation modality and task modality [47]. Such an effect has previously been described in the literature, and is also known as “transfer-appropriate processing approach” [48], referring to the congruence of operations between study and test. The facilitation could lie in the availability of a specific lexicon (although no direct explanation of the concepts was suggested in the verbal descriptions, some of the relevant terms were used, especially labeling the subject and the action). Indeed, the availability of labels could be expected to also have a negative effect on performance, since specific actions and specific subjects are described, which need to be generalized (thus, first inhibited and then possibly substituted if found too specific) in order to give a valid, comprehensive description of the verb meaning. On the other hand, the facilitation might depend on the verbal description “extracting” the relevant information from the scene, omitting very specific details (present in the visual version) which could be disturbing and interfere with the abstraction of the general concept.

In order to test the role of modality congruence in producing the clear advantage of the verbal condition, a second experiment was thus designed, where the output modality was changed from verbal to visual. The results of the second experiment did not confirm a general advantage of the verbal presentation modality for the processing of newly introduced concepts. By contrast, responses turned out to be faster after visual than after verbal presentation. These findings support the hypothesis that congruence between presentation modality and response modality is a facilitating factor. This is in line with studies suggesting an advantage of the visual modality for long-term memorization [39,40]. Moreover, this result could be linked to theories that emphasize the congruence of operations between study and test, such as the so-called “transfer-appropriate processing approach” [48].

The two experiments have shown that there is a developmental pattern in the acquisition of new concepts: in younger children the verbal modality seems to be preferred to learn new concepts, while in older children visual information seems to facilitate concept learning. This developmental pattern of acquisition may be due to the fact that verbal learning is more explicit than visual learning which requires one to infer meaning through an association between image and language competence, an ability that is consolidated over time.

Experiment 1 compared children’s performances in explaining the meaning of a newly presented concept after verbal and visual presentation, and revealed an advantage of the verbal modality. The time-course of iconicity in the two modalities was quite similar. This result supports the idea that language is the main source of information when organizing cues into meaningful frameworks [49,50] and that when children are exposed to a new word, they can comprehend its meaning by analyzing linguistic information, inferring it both from syntactic structure and semantic context [13,14,15,16,17,19,51].

An interesting interaction emerges from the distinction between verbs referring to mental contents and verbs referring to actions, where the latter, but not the former, seem to profit more from verbal than from visual presentation. These rather surprising findings, quite different from those described by [9], remind us that the distinction between mental and action verbs is not an absolute one (in the present study, mental verbs were also at an intermediate level of abstractness, linking mental processes to concrete or specific situations) and that other variables are likely to modulate the ways these concepts are acquired (see [52]). Indeed, some recent work suggests that the acquisition of mental verbs is facilitated by verbal explanations, but this refers to the context of parent–child interaction [9,10,53], which is very different from the school context of the present study. Other, more articulated explanations may also be put forward. Among others, mental verbs in Gleitman and colleagues’ studies were belief verbs introducing complement clauses (e.g., think, believe, etc.), whereas in the present study they were intransitive verbs including reference to the object (e.g., think about sweets, dream about a book). It is therefore possible that the syntactic structure of belief verbs (the complement clause) is the crucial base of the advantage of the verbal modality described in the literature, providing a relevant clue as to the nature of the verb itself (see [10,52,53]). When this specific frame is absent, as in the present study, the advantage of the verbal modality could be lost. The implication would be that the special status of mental verbs derives not from their being more abstract, but from their syntactic peculiarities, as also described by [19], highlighting that transitive, but not intransitive structures, facilitate syntactic bootstrapping. A further conclusion we may drive from the results of the present study is that this distinction between transitive and intransitive constructions may be crucial not only in English, but also in the case of a morphologically rich language like Italian, and also with school-age children. Furthermore, the different results obtained in other studies (e.g., [9]) concerned verbs that could be traced back to already existing verbs: this implies that “grasping the concept” consisted in building a new link to an existing lexical entry in the subject’s vocabulary. Conversely, in the present study no specific existing label (at least, no single label) could describe the newly introduced concepts, so that a completely new node needed to be built in the lexical as well as in the conceptual network. This construction may call on nonverbal functions even in the case of so-called mental verbs, thus reducing the advantage of the verbal presentation modality. Finally, it could be observed that the visual representation of mental verbs in our experiment is facilitated by the use of symbolic tools such as bubbles depicting the content of the actor’s thoughts or wishes. Without the use of such symbolic devices, which are very familiar to children of this age range but could not be employed with younger children, grasping of the meaning of the mental verbs could have been harder. It should be acknowledged, however, that the symbolic devices mentioned above are very representative of the embodied cognition perspective (where the mind is represented as a physical container such as the bubble, see [25,26,27,28]), so the theoretical frameworks are not violated by the experiment. Additionally, it should be acknowledged that the specific type of visual–perceptual representation in the visual condition, which is a static one (pictures), while more dynamic representations such as videos, better suited to show the temporal development of action verbs, might have produced different results. At the very least, thus, the present results allow one to rule out that it is the presence of mental verbs (i.e., of the stimuli occupying the more abstract positions in the concrete-abstract continuum) to determine an advantage of the verbal modality in Experiment 1.

As to the results of Experiment 2, which did not show the same advantage for verbal presentation as Experiment 1, it is important to point out that the visual presentation increases speed in responding, but response accuracy is similar after visual and verbal presentation. Thus, the advantage of the visual modality for response speed in Experiment 2 may suggest that modality congruence could affect response speed due to a fast available mapping between the two representations (from the learning phase and from the response phase) to be compared [47,54], but be irrelevant to the quality of concept learning (accuracy). Indeed, a similar asymmetry has been described for memory performance by [55], who found that only aurally encoded words showed a higher level of neural pattern reactivation when modality matched, whereas no such effect was observed for visually encoded words, and interpreted this as a probable consequence of dual encoding for visual words (although no image presentation was involved in that case, as well as no learning of new concepts). A different explanation may be that analysis in the visual modality (in this case, in the trace of the pictures previously observed in the learning phase) is intrinsically quicker (irrespectively of modality congruence), since it allows in-parallel, distributed search and analysis of features, whereas analysis in the verbal modality is necessarily serial. According to [56], moreover, verbal labels—also noninformative ones—may even enhance efficiency of visual search. In this perspective, there could be a facilitatory effect from the simultaneous presentation of images and verbal cues (the novel concept label). Alternative interpretations call into play the different processes involved in explicit versus implicit responses (in our case, verbal explanation on the one hand, identification of visual instances of the concept on the other hand; see [57,58]), where verbal and visual processing, respectively, could play the main roles [59]. Indeed, recent views of abstract concept use emphasize the importance of investigating concepts not only in situated action, but in ‘situated interaction’, with participants communicating with each other rather than executing artificial tasks [42,43].

Clearly, the kinds of tasks employed in this study allow the possibility that input in a certain modality is recoded in the complementary modality for ease of processing. In this perspective, any disadvantage of a given modality might derive from the additional processing load needed for “translation” into the other modality (see also [33] for examples from bilingual literacy). Even in this case, however, the interpretation of the results would not change in substance: the modality into which translation occurs is revealed to be the more appropriate for information processing and decision making. In a way, the need to recode from one modality to the other could be seen as a reformulation of the “modality congruence effect”, where two elements are easier to be compared if presented in the same modality [48,60,61]. Also the emergence of a significant age effect in the ability to respond correctly to the negative (but not the positive) instances of a concept suggests that processing complexity is a crucial factor in the whole abstraction mechanism.

### Practical Implications and Possible Applications

The present results have both educational and clinical implications. The implications for educational purposes are straightforward: novel verbs (and concepts) at school-age seem to be learned more accurately through verbal descriptions than through visual scenes. This is partly in conflict with the results of previous studies indicating an advantage for visual representations when focusing on the semantic features of the proposed concepts [39], but in line with many studies showed advantages for multimodal presentation in the learning of new notions (see [62,63]) and in second-language acquisition [33,34,64]. However, such studies did not clearly distinguish between abstract and concrete concepts (an exception is [65], who found that the addition of pictures and gestures facilitated especially the learning of L2 abstract nouns), nor did they isolate single grammatical categories as is proposed here, where verbs are the only target of learning. The present study provides specific suggestions for the teaching of abstract terms, especially expressed through verbs. The main contribution of the present findings seems to be that the verbal modality, certainly the preferred one in school situations, may not be the most efficient one to transmit new concepts that have to be processed quickly and accurately, for instance in scientific disciplines or for problem-solving purposes, although it may be appropriate in humanistic and literary disciplines where verbal descriptions will have to be produced by the pupils.

At the clinical level, these results provide a better insight into mechanisms involved into learning processes and, consequently, into learning or language disorders and disabilities. In fact, understanding the way new concepts and new words are acquired enables us to plan and implement more effective rehabilitation strategies in all the situations where children or adolescents have difficulties learning new vocabulary and forming accurate representations for the new terms. Indeed, educational studies showed that it is the students with lower levels of background knowledge who benefit more from combining verbal and visual information [66]. The present results suggest that the use of verbal descriptions could be the most appropriate to support learning in view of explicit use of the new words; however, pictorial representations, especially in association with verbal labels, could provide a better basis when the emphasis is in quick, more implicit processing of the new term and concept (see also [67]). Particularly interesting is the observation that this advantage also exists when the concepts to be learned concern mental verbs, among the most typical examples of highly abstract concepts.

## 5. Limitations of the Study and Future Perspectives

Limitations of this study that should be considered before generalizing the results include the relatively small sample of participants in Experiment 2, which could have prevented further age-related differences or finer interactions with presentation modality being highlighted. Moreover, no thorough matching of word frequency, AoA (age of acquisition) and familiarity was conducted for the two groups of verb types, namely action verbs and mental verbs, so that, even if verbal descriptions were very simple in all cases, it cannot be ruled out that some of the differences are influenced by the lexical parameters of the words. A further limitation concerns the absence of information about long-term retention and generalization to real-world situations. Future studies should address these issues and possibly come to a more precise operationalization of teaching and training procedures to improve abstract concept learning in school or rehabilitation contexts.

## Figures and Tables

**Figure A1:**
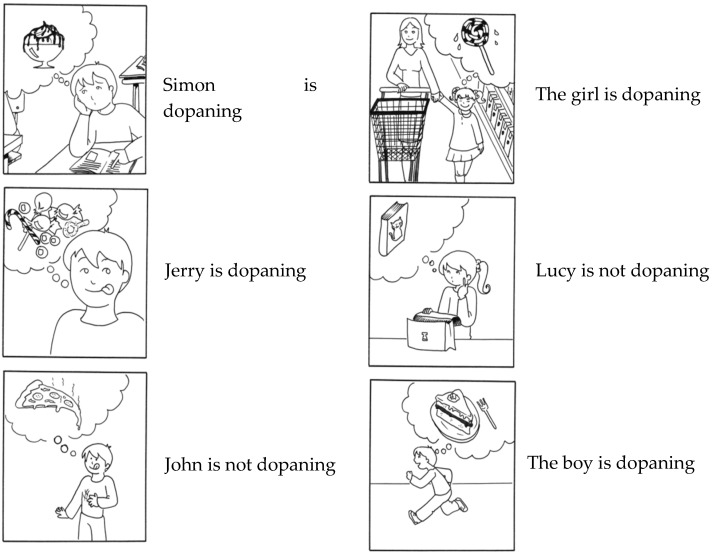
Example of visual presentation: “dopaning” (pseudoword equivalent to “dopanire” = to think about sweets). Six pictures, six statements (auditory presentation).

## Data Availability

The original data presented in the study are openly available in Zenodo, [DOI: 10.5281/zenodo.15277778].

## Appendix A

Complete list of novel abstract concepts employed in the study:

(1) CAGLISTARE: to find a solution to a problematic situation (Mental)
(2) DOPANIRE: to think about sweets (Mental)
(3) DORTARE: to entertain children (Action)
(4) FRINTOLARE: to use objects upside-down (Action)
(5) GINGERE: to help elderly people (Action)
(6) DIFULARE: to fantasize with a book (Mental)
(7) MUPERGERE: to play in a team (Action)
(8) POBEARE: to use an object to reach something (Action)
(9) SOFANIRE: to prevent somebody from doing something dangerous (Mental)
(10) SUNTORARE: to divide things into four parts (Action)

## Appendix B

### Appendix B.2

Example of verbal presentation: “dopaning” (pseudoword equivalent to “dopanire” = to think about sweets). Six verbal descriptions, six statements (auditorily presented).

(1) Simon is at school and there is a book in front of him on his desk. He is thinking about a huge ice cream with hot chocolate on it. Simon is dopaning.
(2) The girl is at the supermarket with her mother, pushing the trolley and she is thinking about buying a lollipop. The girl is dopaning.
(3) Jerry is thinking about a bunch of bonbons. Jerry is dopaning.
(4) Lucy is preparing her bag and she thinks about bringing the cat book with her. Lucy is not dopaning.
(5) John is thinking about a slice of pepperoni pizza. John is not dopaning.
(6) While the boy is running, he thinks about a nice piece of chocolate cake ready to eat. The boy is dopaning.
